# Increased response to oxidative stress challenge in Graves’ ophthalmopathy orbital fibroblasts

**Published:** 2011-10-26

**Authors:** Chieh-Chih Tsai, Shi-Bei Wu, Ching-Yu Cheng, Shu-Ching Kao, Hui-Chuan Kau, Shui-Mei Lee, Yau-Huei Wei

**Affiliations:** 1 Department of Ophthalmology, Taipei Veterans General Hospital and National Yang-Ming University, Taipei, Taiwan; 2 Department of Biochemistry and Molecular Biology, National Yang-Ming University, Taipei, Taiwan; 3 Department of Ophthalmology, Koo Foundation Sun Yat-Sen Cancer Center, Taipei, Taiwan; 4 Department of Medical Research and Education, Taipei Veterans General Hospital, Taipei, Taiwan; 5 Department of Medicine, Mackay Medical College, New Taipei City, Taiwan

## Abstract

**Purpose:**

To investigate whether orbital fibroblasts from patients with Graves’ ophthalmopathy (GO) are more responsive to oxidative stress.

**Methods:**

Lipid peroxidation, oxidative DNA damage, reactive oxygen species (ROS) contents and activities of antioxidant enzymes were measured in cultured orbital fibroblasts from GO patients and age-matched normal controls in response to 200 μM hydrogen peroxide (H_2_O_2_).

**Results:**

GO fibroblasts had increased basal levels of malondialdehyde (MDA), 8-hydroxy 2'-deoxyguanosine, superoxide anions, H_2_O_2_, and manganese-dependent superoxide dismutase (Mn-SOD) activity, as well as decreased glutathione peroxidase (GPx) activity and the ratio between reduced (GSH) and oxidized glutathione (GSSG) compared with the orbital fibroblasts from normal subjects. After treatment of the cells with 200 μM H_2_O_2_, the amplitude of increase in the intracellular levels of MDA (63% versus 26%), H_2_O_2_ (24% versus 13%) and Mn-SOD activity (48% versus 23%) was exaggerated in GO fibroblasts compared with normal controls, respectively. In addition, treatment of GO fibroblasts with 200 μM H_2_O_2_ led to a dramatic reduction of catalase activity (−59% versus −29%), GPx activity (−56% versus −13%), and GSH/GSSG ratio (−49% versus −21%), respectively.

**Conclusions:**

Elevated ROS and redox imbalance in GO orbital fibroblasts were exacerbated by H_2_O_2_ as a result of exhaustion of GSH and compromise of antioxidant enzymes. Hypersensitivity to oxidative stress of GO orbital fibroblasts may play a role in the pathogenesis of GO.

## Introduction

Graves' ophthalmopathy (GO) is the most common extrathyroidal manifestation of Graves’ disease [1]. Many studies have been launched to unravel the pathogenesis of GO, but a clear and indisputable mechanism of the pathogenesis of the disease has not been elucidated [2,3]. This may be a result of a complex interplay between endogenous and environmental factors. Recently, accumulating evidence has shown that oxidative stress plays an important role in the pathogenesis of GO [4-7]. Increased extracellular levels of reactive oxygen species (ROS)-elicited oxidative damage have been noted in the blood [4], urine [5,6], and fibroadipose tissues [7] from GO patients. It is noteworthy that perturbation of the intracellular oxidant/antioxidant balance can lead to the buildup of ROS, which may accumulate in cells and cause widespread cellular injuries. Hydrogen peroxide (H_2_O_2_) is naturally produced in the human cells during many physiologic and pathological processes and has been widely used as a model pro-oxidant in the study of oxidative stress. We have recently reported that biomarkers of oxidative DNA damage and lipid peroxidation are increased in GO fibroblasts [8]. In the present study, we further evaluated oxidative DNA damage, lipid peroxidation, ROS levels, the capacity of free radical scavengers, and the redox state in cultured GO orbital fibroblasts after exposure to exogenous oxidative stress induced by H_2_O_2_ treatment.

## Methods

### Cell culture

Orbital fibroblast cultures were established from surgical waste of four patients with GO during decompression surgery and from apparently normal orbital tissues in three age-matched patients undergoing surgery for noninflammatory conditions. All were not smokers or ex-smokers. All GO patients achieved stable euthyroidism for at least 6 months before surgery and were in the inactive stage of GO. All patients did not undergo corticosteroid treatment for at least 1 month before surgery. The study was performed according to the tenets of the Declaration of Helsinki and these activities have been approved by the Institutional Review Board of Taipei Veterans General Hospital. Briefly, the orbital tissues were minced aseptically in phosphate-buffered saline (PBS), and then incubated with a sterile solution containing 0.5% collagenase and dispase (Sigma-Aldrich Chemical Co., St. Louis, MO) for 24 h at 37 °C in a humidified chamber filled with 5% CO_2_.  The digested orbital tissues were pelleted by centrifugation at 1,000× g, and then resuspended in DMEM containing 10% fetal bovine serum (FBS) and antibiotics (Biological Industries, Kibbutz Beit Haemek, Israel), which was composed of 100 U/ml penicillin G and 100 μg/ml streptomycin sulfate, respectively. [8,9]. Cultured orbital fibroblasts were used between the 3rd and 5th passages and the cultures at the same passage number were used for the same set of experiments.

### Determination of sublethal dose of H_2_O_2_

To determine the sublethal dose of H_2_O_2_ in orbital fibroblasts, normal and GO orbital fibroblasts were treated with 0, 100, 200, and 400 μM H_2_O_2_, respectively. Cell viability was evaluated by using the AlamarBlue^TM^ cell viability assay system (AbD Serotec Ltd., Oxford, UK) [10]. After treatment of cultured cells in 6-well plate with different concentrations of H_2_O_2_ for 90 min, the cells were washed twice with PBS (pH 7.4) to remove H_2_O_2_ and re-cultured in fresh complete DMEM medium. After 24 h, cells seeded in a 6-well plate were washed with PBS and incubated at 37 °C with a fresh medium containing 1× AlamarBlue^TM^ reagent (the assay medium; Invitrogen Corp., Carlsbad, CA) for 4 h. The fluorescence intensity of the assay medium was measured by the Victor^2^_TM_ 1420 Multilabel Counter (Perkin-Elmer Life Sciences Inc., Boston, MA) with the excitation wavelength at 538 nm and the emission wavelength at 590 nm. The data are expressed as means±SD of the results from three independent experiments. When cells were treated with 100–400 μM H_2_O_2_ for 90 min, cell viability of both normal and GO fibroblasts was reduced in a dose-dependent manner. The difference in cell viability between normal and GO orbital fibroblasts was statistically significant upon treatment with 200 μM H_2_O_2_ (84% versus 60%, p=0.003) and 400 μM H_2_O_2_ (70% versus 41%, p<0.001), respectively. Based on these findings, we decided to treat normal and GO orbital fibroblasts with 200 μM H_2_O_2_ as an exogenous oxidative stress in the following experiments.

### Treatment of orbital fibroblasts with H_2_O_2_

Fibroblasts were grown in a 10 cm^2^ Petri dish till about 80% confluence._._ Normal and GO orbital fibroblasts were treated with 200 μM H_2_O_2_ in Dulbecco’s Modified Eagle Medium (Gibco BRL, Invitrogen, Grand Island, NY) supplemented with 10% fetal bovine serum (Biologic Industries, Kibbutz Beit Haemek, Israel) for a period of 90 min followed by measurements of the biomarkers of oxidative DNA damage and lipid peroxidation, the intracellular ROS levels and activities of antioxidant enzymes. Corresponding normal and GO fibroblasts without H_2_O_2_ treatment were used as controls. After H_2_O_2_ treatment, the cells were washed twice with phosphate-buffered saline (PBS, pH 7.3) to remove H_2_O_2_. The cells were then re-cultured with fresh complete DMEM and further incubated at 37 °C for 24 h before being subjected to further experiments.

### Analysis of DNA damage

DNA damage was evaluated by the 8-hydroxy-2'-deoxyguanosine (8-OHdG) content in total DNA, which was determined by using the 8-OHdG ELISA kit from Japan Institute for the Control of Aging (Fukuroi, Japan) according to the manufacturer’s instruction [11]. Briefly, total DNA from the orbital tissue was isolated by phenol/chloroform extraction with the addition of butylated hydroxyl toluene (BHT, freshly prepared in ethanol).  After precipitation with ice-cold 75% ethanol, the DNA was air-dried and dissolved in distilled H_2_O [12]. The detection range of the 8-OHdG concentration under the assay condition was 0.125–10 ng/ml.

### Analysis of lipid peroxidation products

Lipid peroxidation product, malondialdehyde (MDA), in cultured orbital fibroblasts was measured by a spectrophotometric assay kit (MDA-586, OxisResearch, Inc. Portland, OR) according to the manufacturer’s recommended procedure, which involved the reaction with a chromogenic reagent N-methyl-2-phenylindole (NMPI) to form an intensely colored carbocyanine dye with a maximum absorption at 586 nm. A standard curve was established by using the MDA samples at the concentration range of 0–50 μM and the MDA levels in orbital fibroblasts were normalized by the cell number.

### Determination of intracellular ROS

The intracellular ROS content in orbital fibroblasts was measured by using the fluorescent probes 2’,7’-dichlorofluorescin (DCF, 10 μM) and dihydroethidine (DHE, 10 μM; Molecular Probes, Invitrogen, Eugene, OR). DCF staining was used to measure the intracellular H_2_O_2_ levels and DHE was used for the determination of intracellular superoxide anion (O_2_^.-^) levels. After trypsinization, cells were washed with PBS buffer (pH 7.4) followed by resuspension in 0.5 ml of PBS buffer (pH 7.4), and were subjected to analysis on a flow cytometer (Model EPICS XL-MCL; Beckman-Coulter, Miami, FL). The excitation wavelength was set at 488 nm and the intensity of emitted fluorescence of a total of 10,000 cells was recorded at 530 nm on channel FL1 for DCF and at 585 nm on channel FL2 for DHE. Data were acquired and analyzed by using the Cell Quest software (Becton-Dickinson, Franklin, NJ) and each value of GO orbital fibroblasts is presented as a relative value, which was calculated by taking the intracellular ROS levels of the human fibroblast CCD cell line as 100%. The CCD skin fibroblasts were purchased from ATCC (American Type Culture Collection, Manassas, VA) with an ATCC number of CCD-966SK.

### Assay of the activities of antioxidant enzymes

To determine the enzyme activities of superoxide dismutase (SOD), glutathione peroxidase (GPx) and catalase, 10^6^-10^7^ of confluent cells were washed with ice-cold PBS (pH 7.4) before trypsinization. The cell pellets were resuspended in the lysis buffer containing 50 mM Hepes (pH 7.4), 1 mM EDTA, 0.5 mM EGTA, 1% Triton X-100, and an aliquot of complete protease inhibitors (Roche Molecular Biochemicals, Nutley, NJ). The suspension was incubated at 4 °C for 20 min and was then centrifuged at 10,000× g for 30 min at 4 °C. Catalase activity was determined by monitoring the rate of decomposition of H_2_O_2_ based on the decrease in absorbance at 240 nm. The reaction mixture in 1 ml contained 10 mM H_2_O_2_ and 10–20 μl cell lysate in 50 mM Na^+^/K^+^ phosphate buffer (pH 7.4). The enzyme activity was calculated on the basis of an extinction coefficient of 43.6 M^−1^cm^−1^ for H_2_O_2_ at 240 nm. Total SOD activity was measured by monitoring the rate of reduction of nitroblue tetrazolium (NBT; Sigma-Aldrich, St. Louis, MO) according to the method developed by Spitz and Oberley [13]. Mn-SOD activity was differentiated from Cu,Zn-SOD by its resistance to NaCN. In the absence of NaCN, total SOD activity was measured and the Mn-SOD activity was assayed by monitoring NBT reduction in the presence of 5 mM NaCN. GPx activity was determined by using a coupling assay in which enzyme activity is proportional to the rate of NADPH oxidation indicated by a decrease in the absorbance at 340 nm after addition of *tert*-butyl hydroperoxide according to the GPx-340 assay kit [14].

### Determination of reduced glutathione (GSH) and oxidized glutathione (GSSG)

The ratio of GSH/GSSG in orbital fibroblasts was measured by GSH/GSSG-412 kit (OxisResearch, Inc., Portland, OR) according to the manufacturer’s instruction. The assay was designed by using Ellman’s reagent (5,5′-dithiobis-2-nitrobenzoic acid, DTNB), which reacts with GSH to form a product with a maximum absorbance at 412 nm. GSSG can be recycled into GSH by using glutathione reductase and NADPH (nicotinamide adenine dinucleotide phosphate, reduced form) followed by the reaction with DTNB. A calibration curve of standard GSH was constructed by using the range of 0–3 μM GSH for orbital fibroblasts (10^6^ cells), and the GSH/GSSG ratio was then calculated by using the following equation: Ratio=(total glutathione – 2GSSG)/GSSG.

### Statistical analysis

Comparisons of the values of 8-OHdG, MDA, intracellular ROS levels, activities of antioxidant enzymes, and GSH/GSSG ratio between GO and normal fibroblasts were performed using Student's unpaired *t*-test. The difference between the baseline value and the value of treated orbital fibroblasts within each of the two groups was evaluated with Student's paired *t-*test. Statistical analyses were performed using Stata statistical software (Stata Corp., College Station, TX), and the difference was considered statistically significant when p<0.05.

## Results

### H_2_O_2_-induced changes of oxidative DNA damage and lipid peroxidation in the orbital fibroblasts

Before H_2_O_2_ treatment, the mean basal levels of 8-OHdG and MDA in cultured orbital fibroblasts from GO patients were significantly higher than those of normal controls (Table 1). H_2_O_2_ treatment caused a significant increase in the levels of 8-OHdG and MDA in both groups compared to respective controls (without H_2_O_2_ treatment). It is worth mentioning that the amplitude of increase in MDA in response to H_2_O_2_ treatment was more pronounced in GO orbital fibroblasts than that of the normal controls (63% versus 26%, p=0.029).

**Table 1:** Intracellular levels of 8-OHdG and MDA before and after treatment of orbital fibroblasts with 200 μM H_2_O_2_.

**Oxidative damage**	**Before treatment (mean±SD)**	**After treatment (mean±SD)**	Induction ratio (%)* (mean±SD)	**p-value**
**8-OHdG(ng/mg DNA)**				
Normal	2.75±0.24	3.52±0.20	128±5	0.005
GO	3.72±0.20	5.12±0.32	138±11	0.005
	p=0.002		p=0.213	
**MDA (nmol/10^6^ cells)**				
Normal	219.80±35.67	275.33±40.49	126±7	0.027
GO	325.92±10.95	530.95±62.83	163±20	0.007
	p=0.002		p=0.029	

*Induction ratio=after H_2_O_2_ treatment value /baseline value (%).

### H_2_O_2_-induced changes of ROS and antioxidant enzymes in orbital fibroblasts

The changes in oxidative stress markers and antioxidant enzymes upon treatment of orbital fibroblasts with H_2_O_2_ are shown in Table 2 and Table 3. In the GO orbital fibroblasts groups before H_2_O_2_ treatment, the intracellular levels of superoxide anions (O_2_^.-^), H_2_O_2,_ and Mn-SOD activity were higher, whereas GPx activity was lower, than those of age-matched normal controls. H_2_O_2_ treatment led to significant elevation in the levels of superoxide anions, H_2_O_2,_ and Mn-SOD activity and significant reduction of catalase and GPx activities in both groups compared with respective controls (without H_2_O_2_ treatment). However, the increase in H_2_O_2_ contents (24% versus 13%, p=0.024) and Mn-SOD activity (48% versus 23%, p<0.001), as well as the decrease of catalase (−59% versus −29%, p<0.001) and GPx (−56% versus −13%, p<0.001) activities were more pronounced in GO orbital fibroblasts compared with those in normal controls.

**Table 2:** Intracellular levels of reactive oxygen species in orbital fibroblasts before and after treatment with 200 μM H_2_O_2_.

**ROS levels**	**Before treatment (mean±SD)**	**After treatment (mean±SD)**	Induction ratio (%)* (mean±SD)	**p -value**
O2.- (Relative ratio**)				
Normal	94.29±1.90	102.22±3.47	108±3	0.039
GO	105.54±3.45	118.87±2.92	113±2	<0.001
	p=0.004		p=0.055	
H_2_O_2_ (Relative ratio**)				
Normal	105.18±1.79	118.81±0.76	113±2	0.007
GO	123.19±5.84	152.97±6.61	124±6	0.002
	p=0.004		p=0.024	

*Induction ratio=O_2_^.-^ or H_2_O_2_ value after H_2_O_2_ treatment / baseline value (%). **Each measurement of cultured orbital fibroblasts was presented as a relative value, which was calculated by taking the intracellular ROS levels of the human skin fibroblasts CCD cell line as 100%.

**Table 3:** Intracellular levels of antioxidant enzymes in orbital fibroblasts before and after treatment with 200 μM H_2_O_2_.

**Activities of antioxidant enzymes**	**Before treatment (mean±SD)**	**After treatment (mean±SD)**	Induction ratio (%)* (mean±SD)	**p-value**
**Cu,Zn-SOD (μmol/min/mg proteins)**				
Normal	94.93±16.31	100.47±14.15	106±5	0.132
GO	86.33±13.02	84.14±7.57	99±13	0.742
	p=0.470		p=0.393	
**Mn-SOD (μmol/min/mg protein)**				
Normal	65.13±10.68	79.79±11.70	123±4	0.011
GO	165.70±30.19	244.93±45.67	148±5	0.002
	p=0.003		p<0.001	
**Catalase (μmol/min/mg protein)**				
Normal	15.47±1.01	10.98±1.74	71±7	0.012
GO	16.15±1.95	6.66±1.04	41±3	<0.001
	p=0.613		p<0.001	
**GPx (mU/mg)**				
Normal	239.22±23.24	207.63±17.53	87±3	0.024
GO	136.42±6.48	59.91±11.75	44±8	<0.001
	p<0.001		p<0.001	

*Induction ratio=enzyme activity after H_2_O_2_ treatment/baseline value (%).

### H_2_O_2_-induced change of the GSH/GSSG ratio in the orbital fibroblasts

**Figure 1:**
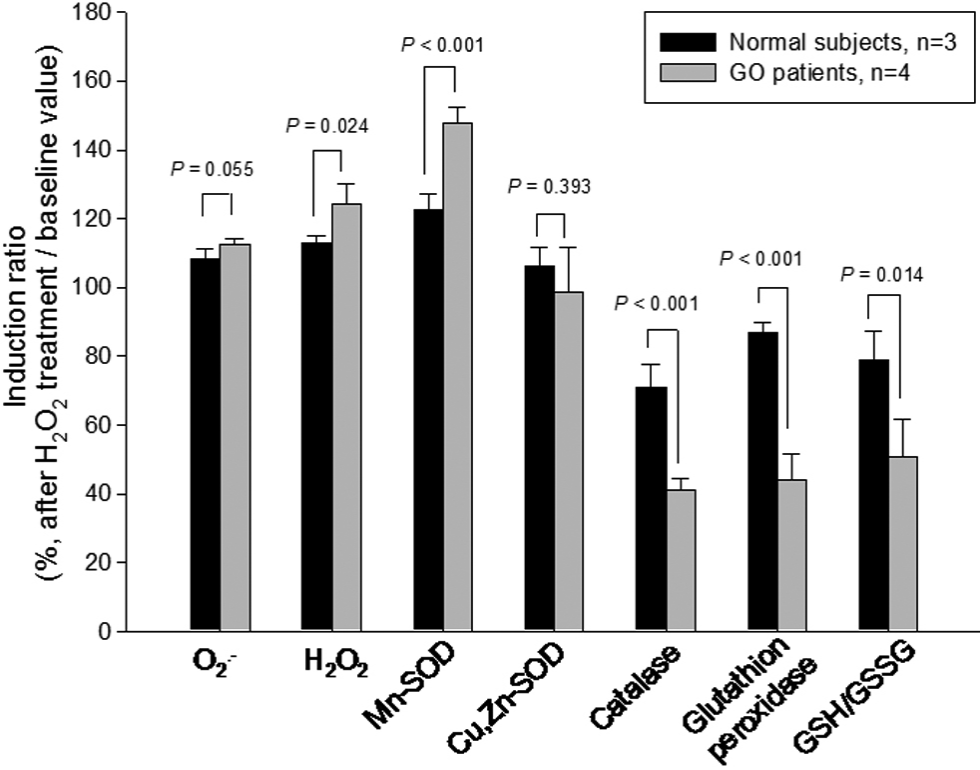
Comparisons of the induction ratio (baseline value divided by after-treatment value) of superoxide anion (O_2_^.-^), H_2_O_2_, Mn-SOD, Cu,Zn-SOD, catalase, glutathione peroxidase, and GSH/GSSG ratio between normal and GO fibroblasts in response to the treatment with 200 μM H_2_O_2_.

The GSH/GSSG ratio was significantly lower in the GO fibroblasts as compared with that of age-matched normal controls (43.15 versus 84.00 p=0.004; Table 4). H_2_O_2_ treatment caused a significant decrease in the GSH/GSSG ratio of the GO orbital fibroblasts (−49%, p=0.008), but not in the controls (−21%, p=0.094). Comparisons of the induction ratio of the superoxide anion (O_2_^.-^), H_2_O_2_, Mn-SOD, Cu,Zn-SOD, catalase, glutathione peroxidase, and GSH/GSSG ratio between normal and GO fibroblasts in response to H_2_O_2_ treatment are summarized in Figure 1.

**Table 4:** GSH/GSSG ratio in orbital fibroblasts before and after treatment with 200 μM H_2_O_2_.

**Redox status**	**Basal treatment (mean±SD)**	**After treatment (mean±SD)**	Induction ratio (%)* (mean±SD)	**p -value**
**GSH/GSSG ratio**				
Normal	84.00±15.21	65.60±6.68	78.94±8.45	0.094
GO	43.15±6.10	21.58±3.58	50.75±10.86	0.008
	p=0.004		p=0.014	

*Induction ratio=GSH/GSSG value after H_2_O_2_ treatment/baseline value (%).

## Discussion

**Figure 2:**
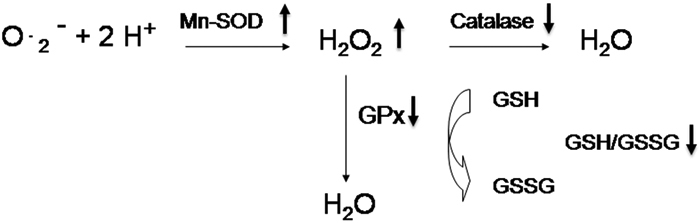
Change of oxidative stress and antioxidant enzymes in cultured GO orbital fibroblasts in response to exogenous H_2_O_2_ . After treatment of orbital fibroblasts with 200 μM H_2_O_2_, the amplitude of increase in H_2_O_2_ and Mn-SOD activity and the amplitude of decrease in catalase activity, GPx activity, and GSH/GSSG ratio were exacerbated in GO fibroblasts as compared with controls.

Orbital fibroblasts, one of the major affected cells in the pathogenesis of GO, are involved in not only the early inflammation process but also the subsequent remodeling process [15]. These orbital fibroblasts have distinct characteristics associated with GO [1-3]. We demonstrated in this study that the intracellular ROS levels and the Mn-SOD activity were higher but the GPx activity was lower in GO orbital fibroblasts. More importantly, we found for the first time that exogenous H_2_O_2_ elicited more pronounced response of ROS metabolism in GO orbital fibroblasts (Figure 2).

MnSOD, GPx, and catalase are major cellular antioxidant enzymes that scavenge ROS such as superoxide anions and H_2_O_2_ and thereby protect cells from oxidative damage. The ROS scavenging function of SOD is effective only when it is followed by the actions of GPx and catalase, because SOD detoxifies superoxide anions to H_2_O_2_, which may become more toxic hydroxyl radicals. Thus, H_2_O_2_ generated by SOD has to be quickly scavenged by catalase or GPx to prevent oxidative damage. GSH, the substrate for GPx, can also counteract the damaging effects of H_2_O_2_. The results of the present study indicate that chronic stress-induced overproduction of ROS was caused by the increase in Mn-SOD activity concomitant with a decrease in GPx activity, thus resulting in increased accumulation of H_2_O_2_ (Figure 2). Moreover, it has been established that the ratio of GSH/GSSG is usually high under normal conditions and reflects the redox status of the cells. The marked decrease of GSH/GSSG ratio and the GPx activity in GO orbital fibroblasts indicate a severe redox imbalance in these cells, which in turn leads to further accumulation of endogenous H_2_O_2_ in the GO orbital fibroblasts.

It is noteworthy that exogenous H_2_O_2_ stimulation further exacerbated the preexisting imbalance of the redox status in GO fibroblasts. Such susceptibility to exogenous oxidative stress was also observed in keratoconus corneal fibroblasts and RECQL4-deficient fibroblasts from patients with Rothmund-Thomson syndrome [16,17]. Increased stress-induced generation of ROS may cause more oxidative damage including oxidative DNA damage and lipid peroxidation, which could explain in part the previous observations of elevated oxidative stress parameters in the plasma [4] and urine [5] of GO patients. These results support our hypothesis that oxidative stress contributes to the pathogenesis of GO. On the other hand, one of our previous studies suggested that increased oxidative DNA damage in GO patients was correlated with their clinical evolution, especially the inflammation activity [5]. In addition, it has been pointed out that H_2_O_2_ can induce gene expression of pro-inflammatory cytokines such as IL-1β and TNF-α [18], which play a crucial role in the development of GO [19]. Taken together, we suggest that the increase in oxidative stress play a role in the pathogenesis of GO, especially in the inflammatory process.

It has been reported that ROS, especially H_2_O_2_ and superoxide anions, are associated with the cellular proliferation of many cell types including fibroblasts [20], which is a key pathological feature in the overt manifestation of GO [21]. Burch et al. [22] demonstrated that superoxide anions, generated by using the xanthine oxidase/hypoxanthine system, could induce the cellular proliferation of cultured GO orbital fibroblasts in a dose–response manner. In addition, at low concentrations H_2_O_2_ (usually under submicromolar concentrations) has been found to stimulate proliferations of a variety of cell types including fibroblasts [23,24]. Heufelder et al. [25] demonstrated that the expression of heat shock protein-72 (HSP72), an important factor in site-directed autoimmune response of GO, was strongly enhanced in GO fibroblasts by H_2_O_2_. In combination with previous observations, the elevated levels of superoxide anions and H_2_O_2_ in GO orbital fibroblasts not only indicate the imbalance of the oxidant/antioxidant status in these cells but also further substantiate the important role of ROS in the development and progression of GO.

Up to the present, highly effective therapeutic strategies for GO have remained elusive. Systemic corticosteroid and radiotherapy remains the mainstay treatment for GO. In a small case series, oral antioxidants showed encouraging results in the treatment of mild and moderately severe GO [26]. One of our recent studies revealed that systemic corticosteroids are effective in the reduction of both the clinical evolution and oxidative DNA damage in patients with active GO [6]. However, the oxidative DNA damage was slightly reversed after withdrawal of corticosteroids. Besides, the use of corticosteroid is often limited to 3–5 months due to side effects, and disease often recurs after cessation of treatment. Therefore, a supplementation of certain antioxidants may be beneficial for GO patients, especially in those subjected to oxidative stress or after withdrawal of corticosteroids. In addition, a biphasic effect of ROS on cell proliferation has been observed, in which low concentrations of ROS induced growth but higher concentrations cause oxidative damage to DNA, proteins and lipids which could potentially lead to apoptosis or necrosis [27]. Though the exact mechanism is still unclear, the ROS at submicromolar levels appear to act as second messengers that are capable of promoting growth of human cells in culture [28]. Our current findings of increased intracellular ROS in GO orbital fibroblasts may suggest that early blockage of ROS formation might be potentially beneficial for GO patients. However, more studies are warranted to provide more information about the effect of antioxidants supplementation in the treatment of GO.

In conclusion, this study demonstrated that GO fibroblasts have exaggerated response to oxidative stress challenge along with imbalance of the activity levels of antioxidant enzymes. As the important target cells in the development of GO, the orbital fibroblasts from GO patients exhibited important differences in their biochemical characteristics and phenotypes that led us to contend that oxidative stress plays an important role in the pathogenesis of GO.
